# Visceral Adipose Tissue Shows Stronger Links to both Chronological and MRI Predicted Brain Age Compared to Subcutaneous Adipose Tissue

**DOI:** 10.1002/alz70856_106573

**Published:** 2026-01-10

**Authors:** Cyrus A. Raji, Somayeh Meysami, Soojin Lee, Saurabh Garg, Nasrin Akbari, Rodrigo Solis Pompa, Ahmed Gouda, Thanh Duc Nguyen, Saqib Basar, Yosef Gavriel Chodakiewitz, David A. Merrill, Amar Patel, Daniel J. Durand, Sam Hashemi

**Affiliations:** ^1^ Mallinckrodt Institute of Radiology, Washington University, St. Louis, MO, USA; ^2^ Pacific Brain Health Center, Pacific Neuroscience Institute and Foundation, Santa Monica, CA, USA; ^3^ Saint John's Cancer Institute at Providence Saint John's Health Center, Santa Monica, CA, USA; ^4^ Vigilance Health Imaging Network Inc., Vancouver, BC, Canada; ^5^ Prenuvo, Palo Alto, CA, USA

## Abstract

**Background:**

Brain age – an image derived measure from structural brain images on T1 weighted scans may reveal information on Alzheimer's risk. We have previously shown that increased abdominal adipose tissue relates to brain atrophy. We evaluated the links between abdominal adipose tissue and brain age.

**Method:**

A total of 1,164 healthy participants from four sites (mean chronological age 55.17 ± 12.37 years, 52% women; 48% men; 39% non‐white) were scanned on 1.5T MR machines with a whole‐body protocol. Whole body sequences utilized in the quantitative analyses of abdominal fat were coronal T1 were used to segment visceral adipose tissue (VAT) and subcutaneous adipose tissue (SAT) segmentation. In this process, a nnU‐Net model was used for fully supervised segmentation and ITK‐SNAP was used for manual annotation. Brain age was computed using a regression‐based 3D Simple Fully Convolutional Network. The model was trained on in‐house T1‐weighted MRI scans collected from 5,500 healthy individuals, aged 18 to 89 years. Brain age gap (BAG) was computed by subtracting chronological age from brain age. Bivariate correlations between VAT and SAT to chronological and brain age were done with partial correlations adjusted for sex with brain age. VAT and SAT were normalized to total abdominal body fat volume. Chronological age was not adjusted for in brain age models to avoid collinearity.

**Result:**

Mean brain age exceeded chronological age (mean brain age = 56.04 ± 12.65, mean BAG = 0.69) and were highly correlated (r=0.94, *p* <.001). VAT and SAT were positively related to increased chronological age (VAT: r=0.2780, *p* = 5.477e‐20; r=0.0924, *p* = 0.002817) and increased brain age (VAT: r=0.2806, *p* = 2.42e‐20; SAT: r=0.0947, *p* = 0.002189) with VAT being more closely linked to age than SAT. This did not change when adjusting for sex in separate partial correlations between VAT and SAT for brain age (VAT: rp = r=0.2948, *p* = 2.247e‐22; SAT: r=0.1070, *p* = 0.0005353). No statistically significant link was noted with VAT, SAT, and BAG.

**Conclusion:**

Both VAT and SAT are linked to chronological and brain age with VAT being more strongly linked. VAT may be a key target for modifying brain age and Alzheimer's risk.

